# Bone Marrow p16^INK4a^-Deficiency Does Not Modulate Obesity, Glucose Homeostasis or Atherosclerosis Development

**DOI:** 10.1371/journal.pone.0032440

**Published:** 2012-03-05

**Authors:** Kristiaan Wouters, Céline Cudejko, Marion J. J. Gijbels, Lucia Fuentes, Kadiombo Bantubungi, Jonathan Vanhoutte, Rebecca Dièvart, Charlotte Paquet, Emmanuel Bouchaert, Sarah Anissa Hannou, Florence Gizard, Anne Tailleux, Menno P. J. de Winther, Bart Staels, Réjane Paumelle

**Affiliations:** 1 Univ Lille Nord de France, Lille, France; 2 Inserm, U1011, Lille, France; 3 Université Droit et Santé de Lille, Lille, France; 4 Institut Pasteur de Lille, Lille, France; 5 Departments of Molecular Genetics and Pathology, Cardiovascular Research Institute Maastricht, Maastricht University, Maastricht, The Netherlands; 6 Department of Medical Biochemistry, Academic Medical Center, University of Amsterdam, Amsterdam, The Netherlands; University of Tor Vergata, Italy

## Abstract

**Objective:**

A genomic region near the CDKN2A locus, encoding p16^INK4a^, has been associated to type 2 diabetes and atherosclerotic vascular disease, conditions in which inflammation plays an important role. Recently, we found that deficiency of p16^INK4a^ results in decreased inflammatory signaling in murine macrophages and that p16^INK4a^ influences the phenotype of human adipose tissue macrophages. Therefore, we investigated the influence of immune cell p16^INK4a^ on glucose tolerance and atherosclerosis in mice.

**Methods and Results:**

Bone marrow p16^INK4a^-deficiency in C57Bl6 mice did not influence high fat diet-induced obesity nor plasma glucose and lipid levels. Glucose tolerance tests showed no alterations in high fat diet-induced glucose intolerance. While bone marrow p16^INK4a^-deficiency did not affect the gene expression profile of adipose tissue, hepatic expression of the alternative markers Chi3l3, Mgl2 and IL10 was increased and the induction of pro-inflammatory Nos2 was restrained on the high fat diet. Bone marrow p16^INK4a^-deficiency in low density lipoprotein receptor-deficient mice did not affect western diet-induced atherosclerotic plaque size or morphology. In line, plasma lipid levels remained unaffected and p16^INK4a^-deficient macrophages displayed equal cholesterol uptake and efflux compared to wild type macrophages.

**Conclusion:**

Bone marrow p16^INK4a^-deficiency does not affect plasma lipids, obesity, glucose tolerance or atherosclerosis in mice.

## Introduction

Genome-wide association studies (GWAS) identified a linkage disequilibrium between several single nucleotide polymorphisms (SNPs) in non-coding regions of the human chromosome 9p21 and the risk for type 2 diabetes [1] and atherosclerotic vascular disease [2]. The coding sequences closest to this locus include CDKN2A (coding for the cyclin-dependent kinase [CDK] inhibitors p16^INK4a^ and its alternative reading frame transcript variant p14^ARF^), CDKN2B (coding for the CDK inhibitor p15^INK4b^) [3], and ANRIL, an antisense non-coding RNA [4] partially overlapping the high risk interval [5]. The gene products of the CDKN2A/B locus are known tumor suppressors which function as cell cycle inhibitors [3]. ANRIL is implicated in CDKN2A [6] and CDKN2B [7] silencing and ANRIL expression strongly correlates with the identified risk-associated SNPs [8]. In line, several of these SNPs correlate with p16^INK4a^ and p15^INK4b^ expression levels in peripheral blood cells [8], suggesting a role for the gene products of the CDKN2A/B locus in the found genetic association.

Interestingly, deletion of a region of mouse chromosome 4, orthologous to the human 9p21 risk interval, was shown to increase body weight, further linking this region with obesity and metabolic risk [9]. Atherosclerosis formation induced by a cholate-containing diet was not affected. However, these mice were not crossed in an atherosclerosis-prone genetic background [9], making data interpretation very difficult with respect to atherosclerosis. Using a series of subcongenic mouse strains, Kuo et al. recently linked this genomic region to decreased p16^INK4a^ and p19^ARF^ (the murine variant of p14^ARF^) gene expression in macrophages and monocytes and to atherosclerosis formation in the atherosclerosis-prone low density lipoprotein receptor-deficient (ldlr^−/−^) background [10]. Additionally, p19^ARF^-deficiency has been shown to be pro-atherogenic in an apolipoprotein E-deficient (apoe^−/−^) background [11]. Although the latter study did not clearly identify the cell type involved, the study of Kuo et al. showed that bone marrow-specific reduced CDKN2A expression (including p16^INK4a^ and p19^ARF^) is required and sufficient to accelerate atherosclerosis formation in ldlr^−/−^ mice [10]. However, the individual contribution of p16^INK4a^ remains unknown.

Bone marrow cells give rise to immune cells, which play a crucial role during the initiation and propagation of type 2 diabetes and atherosclerosis. A key event in type 2 diabetes and atherosclerosis is the recruitment of inflammatory cells to, respectively, adipose tissue (AT) [12] and the neo-intima of large arteries [13]. p16^INK4a^ is expressed in macrophages of human atherosclerotic plaques, where it correlates with the expression of the macrophage marker CD68 and with tumor necrosis factor (TNF) [14]. Recently, we showed that p16^INK4a^-deficiency in murine bone marrow-derived macrophages inhibits inflammatory JAK2-STAT1 signaling, promoting the polarization into alternatively activated (M2) macrophages [15], which have been shown to protect against the development of obesity-induced glucose intolerance and insulin resistance [16]. In addition, p16^INK4a^ plays a role in T-lymphocytes, an immune cell type involved in type 2 diabetes [17] and atherosclerosis [13] development, by inhibiting their activation-induced proliferation [18] and by inducing apoptosis [19]. Moreover, we have recently discovered that p16^INK4a^ expression levels play a role in determining the inflammatory phenotype of adipose tissue macrophages (ATM) [20].

Given the role of immune cell p16^INK4a^, given the association of the CDKN2A locus to cardiovascular disease and type 2 diabetes and given the effects of bone marrow CDKN2A-deficiency on atherosclerosis development, we investigated the contribution of bone marrow p16^INK4a^-deficiency to the development of obesity, glucose intolerance and atherosclerosis in appropriate mouse models.

## Materials and Methods

### Mice and diets

Low density lipoprotein receptor-deficient (ldlr^−/−^) acceptor mice were house-bred and C57BL/6J acceptor mice were purchased from Charles River. All protocols were conducted with the approval of the ethical review boards of the Pasteur Institute, Lille, France (CEEA 13/2010) and Maastricht University, Maastricht, The Netherlands (DEC 2007-117). p16^INK4a^-deficient mice on the C57BL/6J background (>97%) were kindly provided by P. Krimpenfort.

For metabolic experiments, chimeric male C57/BL/6J mice were given a high fat diet (HFD) containing 20% lard and 35% sucrose (Ssniff, Germany). For atherosclerosis experiments, chimeric female ldlr^−/−^ mice were given western diet containing 21% milk butter and 0.2% cholesterol (Safe, France).

After the dietary period, blood samples were collected be retro-orbital puncture and animals were sacrificed by cervical dislocation and organs were snap frozen for further analysis.

### Biochemical analysis

Plasma levels of total cholesterol (TC), triglycerides (TG) and high density lipoprotein cholesterol (HDL-C) were measured using commercially available kits (BioMérieux). Non-HDL-cholesterol (N-HDL-C) was calculated by subtraction of HDL-C from TC. Alanine aminotransferase (ALAT) (Biolabo), aspartate aminotransferase (ASAT) (Biolabo), insulin (Mercodia) and free fatty acid (Diasys) levels were determined according to the manufacturer's instructions.

### Bone marrow transplantation (BMT)

8 week-old male C57BL/6J or female ldlr^−/−^ mice were lethally irradiated (8 Gy) and tail vein injected the next day with 10^7^ bone marrow cells isolated from 8 week-old p16^−/−^ or p16^+/+^ littermate donor mice. Mice received autoclaved acidified water (pH = 2) supplemented with neomycin 100 mg/L (Cat.N1142, Sigma-Aldrich) and polymyxin B sulphate 60000 U/L (Cat.21850029, Invitrogen) 1 week before and 4 weeks after transplantation. Mice were studied 6 weeks post-transplantation allowing complete repopulation by the donor bone marrow. To ensure that donor bone marrow efficiently replaced the resident blood cell population, DNA was extracted from whole blood with an Illustra blood kit (GE Healthcare). PCR was performed with the forward 5′-GCA-GTG-TTG-CAG-TTT-GAA-CCC-3′ and reverse 5′-TGT-GGC-AAC-TGA-TTC-AGT-TTG-3′ primers, yielding products of different lengths depending on the genotype, separated on a 1.5% agarose gel and quantified with the Gel Doc XR system (Bio-Rad). Over 95% of host blood cells were from donor origin. Flow cytometric analysis of blood cell populations was performed as described [21].

### Isolation of the stromal vascular fraction from white adipose tissue (WAT)

AT was cut into small pieces, washed with PBS and digested in Krebs buffer (pH = 7.4) containing collagenase (1.5 mg/ml, Roche Diagnostic) at 37°C during 1 h, filtered through a 200 µm filter (Spectra Mesh nylon filters; Biovalley) and centrifuged at 1500 rpm for 15 min to separate floating adipocytes. After two washes of the adipocyte fraction with PBS, adipocytes were collected and kept at −80°C for DNA and RNA extraction. The Stromal Vascular cell Fraction (SVF) was pelleted and red blood cells were lysed with erythrocyte lysis buffer (NH_4_Cl, 131 mM; NH_4_CO3, 9 mM; EDTA, 1 mM; pH 7.4) for 5 min; the remaining cells were filtered through filter meshes with pore size of 70 µm (Spectra Mesh nylon filters; Biovalley). SVF was subjected to Magnetic-Activated Cell Sorting (MACS) in order to isolate ATM CD11b^+^ cells using CD11b-labelled magnetic beads (Miltenyi Biotec) according to the manufacturer's instructions. The CD11b^−^ fraction was cultured in Preadipocyte Basal Medium (PBM, Promocell) for 24 h to eliminate cell types other than preadipocytes. Only adherent cells were used for RNA and DNA extraction. RNA extraction was performed using RNeasy kits (Qiagen) and DNA extraction with Nucleospin Tissue columns (Marchery-Nagel) according to the manufacturer's instructions. PCR was performed as described for blood chimerism.

### RNA quantification by real-time PCR

Tissue RNA was isolated using the guanidinium thiocyanate (GSCN)/phenol/chloroform extraction method and 1 µg mRNA was reverse transcribed using the SuperScript First-strand Synthesis System for Reverse transcription (Life Technologies, Gaithersburg, MD, USA). Reverse transcribed cDNAs were quantified by SYBR green-based real-time PCR using specific oligonucleotides on a Mx3000 apparatus (Stratagene, La Jolla, CA). mRNA levels were normalized to cyclophilin expression as internal control.

### Glucose Tolerance Tests (GTT)

Blood samples were collected from the tail vein at 0, 5, 15, 30, 60, 90 or 120 min after a bolus glucose (1 g/kg) injection (after fast 6 hours of fasting) and glycemia was measured using a Glucometer (Accu-Check active®, Roche Applied Science). To avoid time-dependent effects, GTT were performed at 2 different time points, i.e. after 16 and 20 weeks of high fat feeding. To circumvent influences of glucose injection on hepatic gene expression, animals were sacrificed 2 weeks after the last GTT, i.e. after 22 weeks of HFD.

### Macrophage cholesterol assays

Bone marrow-derived macrophages (BMDM) were obtained from femoral and tibial bone marrow suspensions plated at 10×10^6^ cells in 10 cm plates and differentiated in bone marrow medium (RPMI 1640 containing Hepes 25 mM supplemented with 10% low endotoxin fetal bovine serum, 15% L929-conditioned medium, 2 mM L-glutamine, 1 mM gentamycine). Cholesterol esterification was assessed by measuring the incorporation of [14C]-oleate into cholesteryl esters after incubation with acetylated (Ac) LDL (50 µg/mL) for 24 and 48 hours. After the cholesterol-loading period, cholesteryl ester formation was measured as described [22]. Cholesterol efflux to HDL was measured after macrophage cholesterol-loading with [3H]cholesterol-AcLDL (50 µg/mL) for 48 h as described [22].

### Atherosclerotic Lesion analysis

Mice were euthanized by cervical dislocation and the heart of each animal was rinsed with PBS and fixed with 4% phosphate-buffered paraformaldehyde (pH = 7.4). Serial 10-µm-thick sections were cut between the valves and the aortic arch and lipid deposition was quantitatively analyzed by Oil red-O staining. Toluidine staining and Sirius red staining were performed and plaque morphology and stability were scored by a blinded animal pathologist as described [21]. Immunohistochemical analysis of TNF and MCP1 was performed using specific antibodies (Santa Cruz Biotechnology), followed by detection with biotinylated secondary antibodies and streptavidin-horseradish peroxidase. Immunostainings were visualized using the DAB substrate-chromogen system (DAKO Corporation, Carpinteria, Calif). Images were captured using a JVC 3-charge-coupled device video camera. Sections were analyzed using the computer-assisted Quips Image analysis system (Leica Mikroskopic und System GmbH, Wetzlar, Germany).

### Statistical analysis

Groups were compared using 2-tailed non-paired *t*-tests or 2-way ANOVA using GraphPad Prism software. *In vitro* data are expressed as means ± SD and *in vivo* data are expressed as means ± SEM.

## Results

### Bone marrow p16^INK4a^-deficiency does not affect high fat diet-induced obesity or glucose intolerance

**Figure 1:**
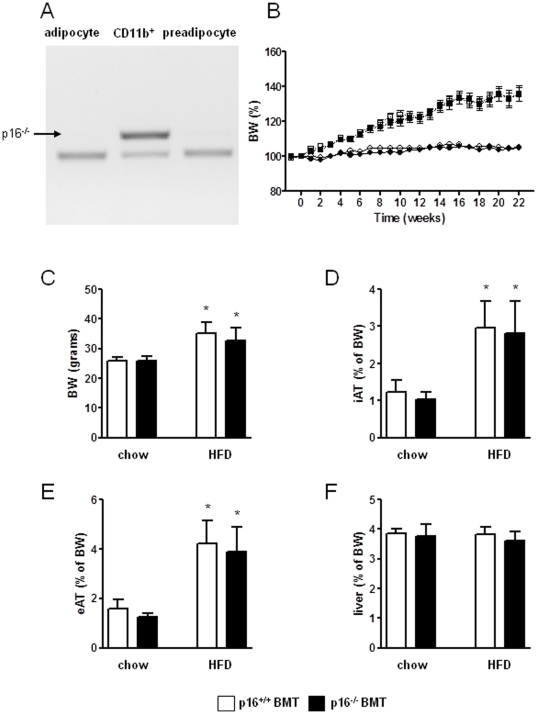
Bone marrow p16^INK4a^-deficiency does not modulate HFD-induced body weight gain. A. Genotype (arrow shows the p16^−/−^ DNA construct) of adipocytes, CD11b^+^ cells (macrophages) and preadipocytes isolated from AT of mice transplanted with p16^−/−^ bone marrow after 16 weeks of HFD (22 weeks post-transplantation). B. Body weight was monitored of mice transplanted with p16^+/+^ (p16^+/+^ BMT) (chow n = 9, open circles; HFD n = 12, open squares) or p16^−/−^ (p16^−/−^ BMT) (chow n = 8, closed circles; HFD n = 12, closed squares) bone marrow and were sacrificed after 22 weeks of dietary treatment. Body weight (C), inguinal AT (D), epidydimal AT (E) and liver (F) weights were measured. White bars represent p16^+/+^ BMT mice and black bars represent p16^−/−^ BMT mice. Statistical differences are indicated * p<0.05 compared to chow fed animals of the same genotype.

Since we previously observed that p16^INK4^-deficiency modulates the inflammatory phenotype of murine macrophages [15] and since p16^INK4a^ knock-down directs human macrophages toward an ATM-like phenotype [20], we analyzed the contribution of the bone marrow p16^INK4a^-deficiency to diet-induced adipose tissue development and glucose intolerance in mice. C57BL/6 mice were transplanted with either wild-type (p16^+/+^ BMT) or p16^INK4a^-deficient (p16^−/−^ BMT) bone marrow. After 6 weeks of recovery, over 95% of blood cells were from donor origin (data not shown) and blood immune cell composition was similar between the genotypes (data not shown). Importantly, after 16 weeks of HFD feeding, the presence of donor cells in the macrophage (CD11b^+^) fraction of adipose tissue (AT) was over 85% while neither mature adipocytes, nor preadipocytes were from donor origin (Fig. 1A). The animals were fed a HFD during 22 weeks and body weight and blood parameters were monitored. Food intake was equal during the dietary period (data not shown). No difference was observed in body weight gain between p16^+/+^ BMT and p16^−/−^ BMT mice either on normal chow diet or on a HFD (Fig. 1B). At the end of the dietary period, neither body weight (Fig. 1C), inguinal AT (iAT) (Fig. 1D), epidydimal AT (eAT) (Fig. 1E) nor liver weight (Fig. 1F) were different between the genotypes. Plasma levels of glucose, insulin, free fatty acids (FFA), liver enzymes (ALT/AST) and lipids did not differ between p16^+/+^ BMT and p16^−/−^ BMT mice after 22 weeks on chow or on a HFD (Table 1).

**Table 1:** Plasma parameters.

	p16^+/+^ BMT		p16^−/−^ BMT	
	chow	HFD	chow	HFD
Glycemia (mg/dl)	104±8	154±27***	103±16	140±23*
Plasma Insulin (µg/L)	0.8±0.5	1.59±0.8*	0.66±0.2	1.64±0.7
Plasma FFA (nM)	0.6±0.2	0.61±0.2	0.75±0.2	0.95±0.6
Plasma AST (UI/L)	30±9	32±8	32±5	34±6
Plasma ALT (UI/L)	32±13	31±6	28±3	29±7
Plasma total Cholesterol (mg/dl)	77±6	145±33***	79±7	134±30***
HDL Cholesterol (mg/dl)	50±6	88±23***	51±4	79±21*
non-HDL Cholesterol (mg/dl)	26±6	56±16***	24±10	55±12***
Triglycerides (mg/dl)	76±11	63±12	91±12	72±21

	p16^+/+^ BMT ldlr^−/−^		p16^−/−^ BMT ldlr^−/−^	
	chow	HFD	chow	HFD
Plasma total Cholesterol (mg/dl)	242±30	1135±135***	238±32	1034±144***
HDL Cholesterol (mg/dl)	67±4	103±13***	63±10	107±16***
non-HDL Cholesterol (mg/dl)	172±21	1037±137***	175±31	927±149***
Triglycerides (mg/dl)	124±29	398±105***	124±24	400±91***

Plasma lipids were measured after 22 weeks standard chow or HFD feeding of male p16^+/+^ BMT (chow n = 9; HFD n = 12) or p16^−/−^ BMT (chow n = 8; HFD n = 12) mice. Plasma lipid measurements before (chow) and after (9 weeks) the dietary period in p16^+/+^ BMT ldlr^−/−^ (n = 17) or p16^−/−^ BMT ldlr^−/−^ (n = 16) mice. Values are shown ± standard deviation. Statistical differences are indicated * p<0.05, ** p<0.01, *** p<0.001 compared to chow-fed controls.

**Figure 2:**
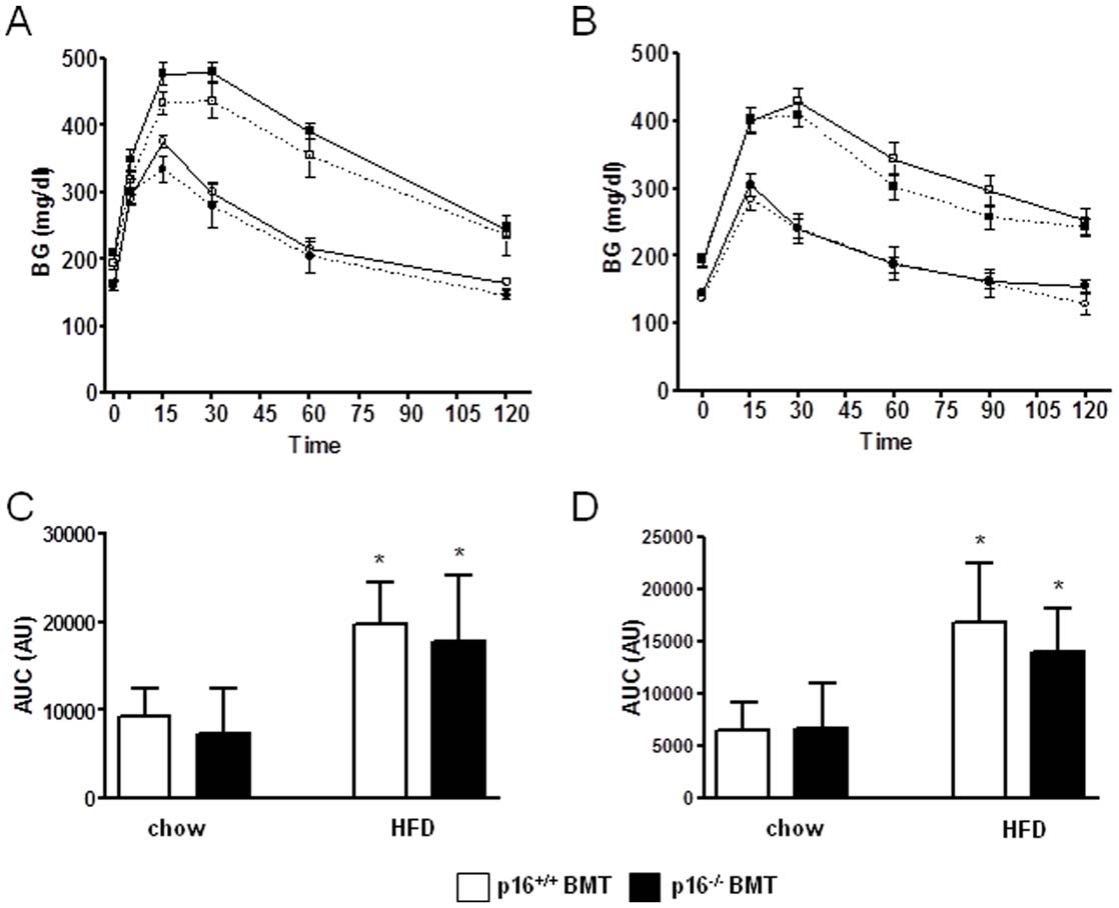
Bone marrow p16^INK4a^-deficiency does not affect HFD-induced glucose intolerance. Glucose tolerance tests (GTT) were performed after 16 and 20 weeks of HFD feeding of male p16^+/+^ BMT (chow n = 9, open circles; HFD n = 12, open squares) or p16^−/−^ BMT (chow n = 8, closed circles; HFD n = 12, closed squares) mice. Blood glucose values were monitored after 1 g/kg glucose injection (A. 16 weeks; B. 20 weeks) and areas under the curve were calculated (C. 16 weeks; D. 20 weeks). White bars represent p16^+/+^ BMT mice and black bars represent p16^−/−^ BMT mice. Statistical differences are indicated * p<0.05 compared to chow fed animals of the same genotype.

**Figure 3:**
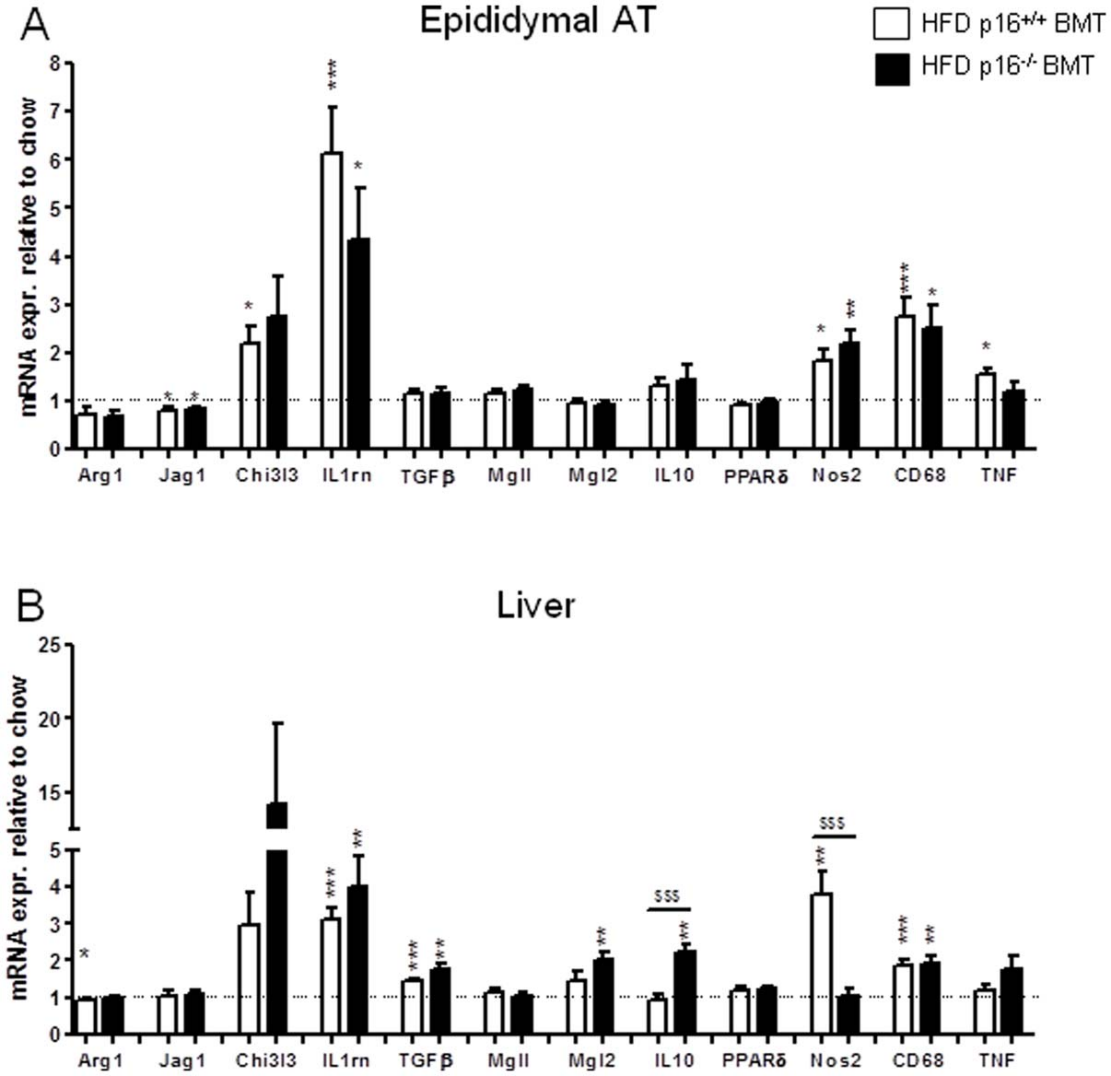
Bone marrow p16^INK4a^-deficiency alters hepatic, but not AT HFD-induced gene expression. p16^+/+^ BMT (chow n = 9; HFD n = 12) or p16^−/−^ BMT (chow n = 8; HFD n = 12) mice were sacrificed after 22 weeks of dietary treatment. Epidydimal AT (A) and liver (B) mRNA were isolated and inflammatory genes were measured by qPCR. Data are expressed relative to low fat chow-fed controls of the same genotype (set at 1, dotted line). White bars represent p16^+/+^ BMT mice and black bars represent p16^−/−^ BMT mice. Statistical differences are indicated * p<0.05, ** p<0.01, *** p<0.001 compared to chow-fed controls; $$$ p<0.001 compared between the HFD-fed groups.

No difference in glucose tolerance was observed between p16^+/+^ BMT and p16^−/−^ BMT mice on chow or after either 16 weeks or 20 weeks of HFD (Fig. 2A–D), time points when at least 85% of ATMs were from donor origin (Fig. 1A). Since p16^INK4a^ alters macrophage polarization status [15] and since obesity is associated with a shift toward classically activated macrophages in AT [23] and in the liver [24], macrophage polarization markers were measured in these organs. The gene expression profile of AT from p16^−/−^ BMT mice revealed no difference in the HFD-induced expression (compared to low fat chow-fed animals as baseline) of either alternatively or classically activated macrophage markers compared to p16^+/+^ BMT mice (Fig. 3A). Interestingly, hepatic HFD-induced expression (compared to low fat chow-fed animals as baseline) of the alternative marker IL10 was higher and the induction of Chi3l3 tended to be higher (p = 0.065) in p16^−/−^ BMT mice. Moreover, Mgl2 was induced in p16^−/−^ BMT mice but not in p16^+/+^ BMT mice (Fig. 3B). Contrary, the induction of the classical marker NOS2 was completely ablated compared to p16^+/+^ BMT mice (Fig. 3B).

Collectively, these data show that bone marrow p16^INK4a^-deficiency does not modulate HFD-induced obesity or glucose metabolism.

### Bone marrow p16^INK4a^-deficiency does not influence atherosclerosis size or lesion composition in hyperlipidemic ldlr^−/−^ mice

Since bone marrow CDKN2A-deficiency in ldlr^−/−^ mice increases atherosclerosis development [10], we investigated the specific contribution of bone marrow p16^INK4a^-deficiency to atherosclerosis development. Atherosclerosis-prone ldlr^−/−^ mice were transplanted with either wild-type (p16^+/+^ BMT ldlr^−/−^) or p16^INK4a^-deficient (p16^−/−^ BMT ldlr^−/−^) bone marrow [25]. The animals were fed a western diet during 9 weeks and physiological parameters and atherosclerosis development were monitored, a time point comparable to the one where the effects of bone marrow CDKN2A-deficiency were observed [10].

Blood cell replacement was higher than 95% and no differences were observed in blood monocyte, neutrophil, B-lymphocyte or T-lymphocyte composition (data not shown). No differences were observed in plasma TC, HDL-C, non-HDL-C or TG between p16^+/+^ BMT ldlr^−/−^ and p16^−/−^ BMT ldlr^−/−^ mice (Table 1).

**Figure 4:**
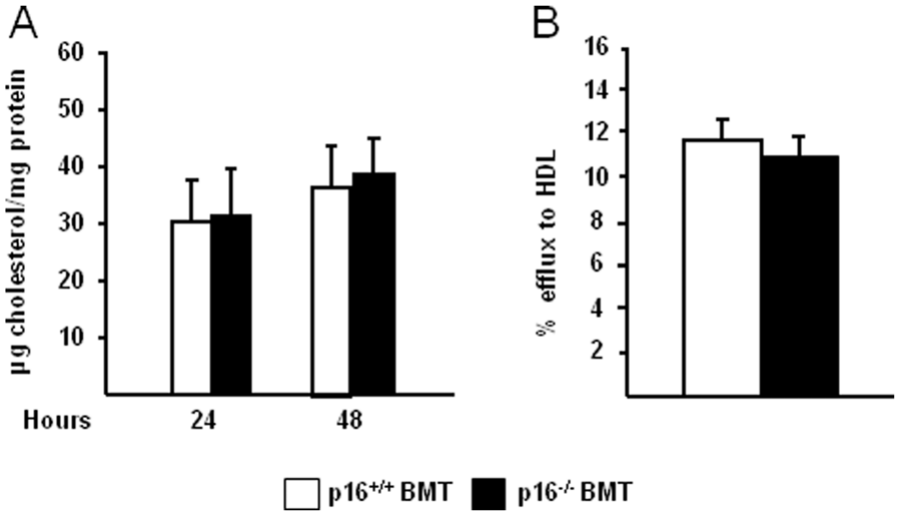
Bone marrow p16^INK4a^-deficiency does not influence macrophage cholesterol metabolism. BMDM from p16^+/+^ and p16^−/−^ mice were cultured and cholesterol uptake (A) and efflux to HDL (B) were measured. White bars represent p16^+/+^ BMDM and black bars represent p16^−/−^ BMDM.

Intracellular cholesterol metabolism in macrophages is a determining factor of plaque initiation and formation [26]. Therefore, since IFNγ influences foam cell formation by diminishing cholesterol efflux [27] and since p16^INK4a^-deficiency alters IFNγ-induced signaling [15], uptake and efflux of cholesterol in wild type and in p16^INK4a^-deficient macrophages were compared. As shown in Fig. 4, cholesterol uptake as well as efflux to HDL in p16^INK4a^-deficient BMDM was equal compared to their wild type counterparts.

**Figure 5:**
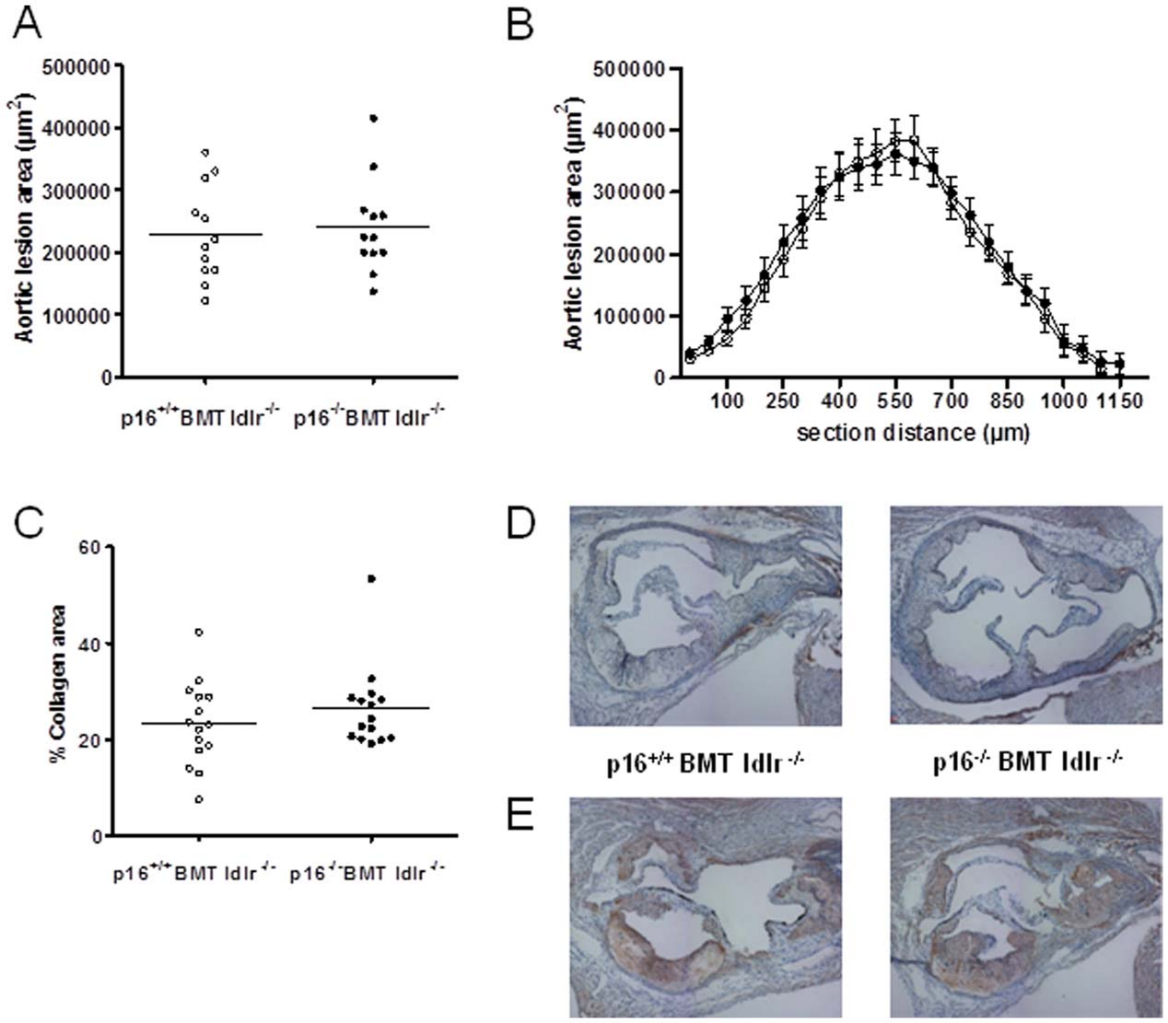
Bone marrow p16^INK4a^-deficiency does not alter atherosclerosis development or plaque phenotype. Ldlr^−/−^ mice transplanted with p16^+/+^ (p16^+/+^ BMT ldlr^−/−^; n = 12) or p16^−/−^ (p16^−/−^ BMT ldlr^−/−^; n = 12) bone marrow were sacrificed after 9 weeks of western diet. (A) Measurement of lesion surface by Oil Red O staining; (B) lesion profile starting from the aortic root; (C) collagen content was calculated by measuring Sirius red staining intensity relative to plaque size; (D) representative pictures of MCP1 immunostaining; (E) representative pictures of TNF immunostaining. Open circles represent p16^+/+^ BMT ldlr^−/−^ mice and closed circles represent p16^−/−^ BMT ldlr^−/−^ mice.

After 9 weeks of western diet, no differences were observed in mean plaque size (Fig. 5A) or in plaque size distribution (Fig. 5B). Moreover, neither plaque Sirius Red-positive collagen content (Fig. 5C), nor the inflammatory phenotype, illustrated by immunohistochemical staining of plaque MCP1 (Fig. 5D) and TNF (Fig. 5E), were affected. Additionally, blind scoring of toluidine-stained sections by an animal pathologist showed no changes in plaque necrosis, foam cell content, monocyte adhesion, neutrophil content or adventitial influx (data not shown).

Collectively, these data show that bone marrow p16^INK4a^-deficiency does not alter atherosclerosis development or plaque phenotype in ldlr^−/−^ mice.

## Discussion

Genetic susceptibility contributes significantly to cardiovascular disease [28], [29] and type 2 diabetes [30] risk. Therefore, the identification of genes and mechanisms behind newly found genetic associations, such as for p16^INK4a^, will provide new insights and therapeutic possibilities for targeting atherosclerosis and type 2 diabetes. The locus coding for p16^INK4a^ has been linked to the risk for type 2 diabetes [1] and atherosclerotic vascular disease [2] and bone marrow-specific heterozygous deletion of CDKN2A (including p16^INK4a^ and p19^ARF^, the murine variant of p14^ARF^) is required and sufficient to accelerate atherosclerosis formation [10]. Therefore, we investigated the specific role of bone marrow p16^INK4a^ on obesity, glucose tolerance and atherosclerosis formation. Our data show that bone marrow p16^INK4a^-deficiency does not alter susceptibility to high fat diet-induced obesity and glucose intolerance or to western diet-induced atherosclerosis development.

Blood cell replacement was efficient as soon as 6 weeks post-transplantation, excluding an ineffective repopulation of the host bone marrow to have influenced our results. Since aging influences p16^INK4a^ expression [31], it is important to note that we used bone marrow from young mice of the same age to avoid age-related effects. Additionally, ATMs were efficiently replaced at the moment of metabolic testing. The observation that p16^INK4a^-deficiency was only found in CD11b^+^ cells but not in pre-adipocytes or in adipocytes contributes to the debate about a possible bone marrow origin of (pre)-adipocytes [32], arguing against such an origin of these cells.

Macrophage inflammatory status is an important determinant of the progression of both atherosclerosis and obesity-induced glucose intolerance. For example, inhibition of IFNγ-induced STAT1 signaling protects against atherosclerosis development [33] and IFNγ is associated with the development of AT in obese patients [34] and diminishes insulin signaling in human adipocytes [35]. We recently discovered that macrophage p16^INK4a^-deficiency inhibits pro-inflammatory JAK2-STAT1 signaling [15]. However, bone marrow p16^INK4a^-deficiency appears insufficient to affect atherosclerosis progression, obesity development or glucose homeostasis in mice. Moreover, despite the effects of p16^INK4a^-deficiency on macrophage polarization status [15], p16^INK4a^-deficiency in bone marrow cells did not influence the inflammatory signature in AT. In the liver, however, we observed higher induction of some alternative activation markers and NOS2 induction was completely inhibited, providing evidence that the anti-inflammatory phenotype of p16^INK4a^-deficient macrophages is, albeit modestly, observed *in vivo*, in line with our previous observations [15].

Our results may reflect that the association of the CDKN2A/B locus with type 2 diabetes and cardiovascular disease depends on gene products other than p16^INK4a^ encoded by this high risk genomic region, such as p19^ARF^ or p15^INK4b^. For example, whole-body deficiency of the other gene product of CDKN2A, *i.e.* p19^ARF^, aggravates atherosclerosis development in apoe^−/−^ mice, although the exact mechanisms and cell types involved *in vivo* remained elusive [11]. Combined with the recently published data showing that bone marrow-specific deletion of the complete CDKN2A locus promotes atherosclerosis development [10], our results exclude the involvement of solely p16^INK4a^. Experiments using p19^ARF^ bone marrow will show whether p19^ARF^ alone or the combined deletion of both CDKN2A gene products in bone marrow-derived cells is required for influencing atherosclerosis progression. However, it has to be taken into account that there is only 50% homology between the human 9p21 and the corresponding murine genomic region [36]. We therefore should be careful when trying to extrapolate the findings of murine studies to humans.

Alternatively, it is possible that p16^INK4a^ is involved in the development of type 2 diabetes and atherosclerosis by acting in other cell types. For example, the CDK4-pRB-E2F1 pathway, which is inhibited by p16^INK4a^, has been shown to control insulin secretion in β-cells [37]. Furthermore, a recent report has shown that bone marrow over-expression of p16^INK4a^ promotes a prothrombotic phenotype in mice [38], indicating that the link between the CDKN2A locus and cardiovascular disease may be primarily related to occlusive vascular events, a parameter that was not investigated in this study. In parallel, the CDKN2A/B locus has been strongly associated with coronary artery calcification [39], which was not investigated in this study. Finally, p16^INK4a^ is also expressed by smooth muscle cells (SMC) and vascular SMC proliferation is an important event during atherosclerosis and vascular occlusion [40]. We have previously shown that p16^INK4a^ plays a role in this process [41], making it feasible that p16^INK4a^ contributes to cardiovascular disease via this mechanism. Since bone marrow p16^INK4a^-deficiency did not affect collagen deposition and since we did not observe any differences with respect to changes in the media of the vessel wall (data not shown), the effects of p16^INK4a^ on vascular remodeling during atherosclerosis and restenosis appear independent of immune cells or other bone marrow-derived cells. Together with the fact that deleting the mouse region orthologous to the human 9p21 risk interval results in increased SMC proliferation *in vitro* [9], these observations point to a major role of p16^INK4a^ in SMC.

In conclusion, our results argue against a major role for p16^INK4a^ in bone marrow cells in the development of obesity, glucose intolerance or atherosclerosis. These observations shed light on the found linkage disequilibrium found in GWAS between these conditions and the CDKN2A/B region. The effects of p16^INK4a^-deficiency on inflammatory phenotype thus appear insufficient to affect atherosclerosis or the development of obesity and glucose intolerance suggesting the involvement of other cell types, other gene products or combinations thereof in the CDKN2A/B genomic region.
